# Large-band-gap non-Dirac quantum spin Hall states and strong Rashba effect in functionalized thallene films

**DOI:** 10.1038/s41598-023-43314-4

**Published:** 2023-09-25

**Authors:** Xiaojuan Liu, Zhijian Li, Hairui Bao, Zhongqin Yang

**Affiliations:** 1 State Key Laboratory of Surface Physics, Key Laboratory of Computational Physical Sciences (MOE), Department of Physics, Fudan University, Shanghai, 200433 China; 2 Shanghai Qi Zhi Institute, Shanghai, 200030 China

**Keywords:** Materials science, Condensed-matter physics, Surfaces, interfaces and thin films

## Abstract

The quantum spin Hall state materials have recently attracted much attention owing to their potential applications in the design of spintronic devices. Based on density functional theory calculations and crystal field theory, we study electronic structures and topological properties of functionalized thallene films. Two different hydrogenation styles (Tl_2_H and Tl_2_H_2_) are considered, which can drastically vary the electronic and topological behaviors of the thallene. Due to the *C*_*3v*_ symmetry of the two systems, the *p*_*x*_ and *p*_*y*_ orbitals at the Γ point have the non-Dirac band degeneracy. With spin–orbit coupling (SOC), topological nontrivial band gaps can be generated, giving rise to non-Dirac quantum spin Hall states in the two thallium hydride films. The nontrivial band gap for the monolayer Tl_2_H is very large (855 meV) due to the large on-site SOC of Tl *p*_*x*_ and *p*_*y*_ orbitals. The band gap in Tl_2_H_2_ is, however, small due to the band inversion between the Tl *p*_*x/y*_ and *p*_*z*_ orbitals. It is worth noting that both the Tl_2_H and Tl_2_H_2_ monolayers exhibit strong Rashba spin splitting effects, especially for the monolayer Tl_2_H_2_ (α_R_ = 2.52 eVÅ), rationalized well by the breaking of the structural inversion symmetry. The Rashba effect can be tuned sensitively by applying biaxial strain and external electric fields. Our findings provide an ideal platform for fabricating room-temperature spintronic and topological electronic devices.

## Introduction

Since Kane and Mele first proposed the quantum spin Hall (QSH) effect in graphene^1,2^, QSH insulators, also known as one type of two-dimensional (2D) topological insulators (TIs), have attracted great attention in condensed matter physics and material science due to their wide potential applications in spintronics and topological quantum computation^3–5^. QSH insulators are characterized by an insulating band gap in bulk and non-dissipative, fully spin-polarized gapless helical edge states at the sample boundary, which are protected by time inversion symmetry. As the first predicted 2D TI, graphene^1^ opens a topologically nontrivial band gap at the Dirac point, with the consideration of the spin–orbit coupling (SOC) effect. However, besides the factor of the light carbon element, the horizontal mirror symmetry in the graphene structure inhibits the first-order SOC effect between the nearest neighbor carbon atoms. Therefore, the weak second-order SOC makes the QSH effect only appear at an unrealistically low temperature^6^. Till now, the experimental observations of quantized Hall conductance through the QSH effect are only reported in few systems including Bismuthene^7^, WTe_2_^8^, and HgTe/CdTe^9,10^ and InAs/GaSb^11,12^ quantum-wells with an ultralow temperature (< 10 K), which limits their tempting applications in room temperature spintronic devices.

To achieve QSH effects at room temperature, 2D materials with strong SOC interactions and large topologically nontrivial band gaps are very desired. Some graphene-like 2D layered materials have been proposed to own QSH effects with relatively large band gaps, including group-IVA monolayers for the low-pucker silicene^13,14^, germanene^13,15^, stanene^16^, and group-VA bismuthene^7^. Based on the existing 2D materials, chemical functionalization has been found being a valid tactic to design the QSH effects with pretty large band gaps^14,16,17^. These researches indicate that two types of schemes may be employed to produce the QSH states with large band gaps. One is designing the materials containing heavy elements which can bring strong SOC effects. Another approach is through chemical functionalization. Their combination should be a more effective tactic.

Besides the groups IVA Pb and VA Bi, group IIIA Tl can also be regarded as a heavy element, whose graphene-like monolayer structure is called thallene^18,19^. The pristine 2D honeycomb-like thallene is a topological trivial semiconductor. The phase transition from a semiconductor to a QSH insulator can be achieved under the application of large biaxial strain and its nontrivial topology comes from a band inversion between *p*_*x/y*_ and *p*_*z*_ orbitals induced by SOC^18^. The thallene monolayer has been successfully prepared in experiments by cooling the 2/3 monolayer of mobile Tl atoms on a single-layer NiSi_2_ atop a Si(111) substrate below ~ 150 K^19^. Compared with the theoretically assumed free-standing thallene, the thallene structure frozen on the NiSi_2_/Si(111) substrate in the experiments undergoes strong tensile strain (~ 27%) and then enters the topological phase^19^. Very recently, high-quality large-scale thallene monolayers with exotic electron bands demonstrating colossal spin-polarization have been fabricated through the decoration of thallene/NiSi_2_ interface by Sn interlayers^20^. Thus, Tl monolayers actually become a new heavy-element material platform, which may be designed to explore the interesting 2D topological electronic states etc.

In this work, based on first-principles calculations, two different configurations of hydrogenated thallene Tl_2_H and Tl_2_H_2_ are built. The electronic structures and topological properties for the two monolayers are systematically studied. Due to the *C*_*3v*_ symmetry of the structures, the Tl *p*_*x*_ and *p*_*y*_ orbitals of the Tl_2_H and Tl_2_H_2_ monolayers form quadratic non-Dirac bands. A topologically nontrivial band gap (up to 855 meV) can be generated by the SOC interaction, giving rise to a non-Dirac quantum spin Hall state in the materials. The variation between the electronic states of the Tl_2_H and Tl_2_H_2_ monolayers can be comprehended through the crystal field splitting and band inversion. Particularly, due to the absence of the structural inversion symmetry, the Tl_2_H and Tl_2_H_2_ monolayers both exhibit marked Rashba spin splitting effect, especially in the monolayer Tl_2_H_2_. The Rashba splitting can be tuned sensitively by applying biaxial strain and external electric fields, beneficial to spintronic applications.

## Results and discussion

### Crystal structures and stability

**Figure 1:**
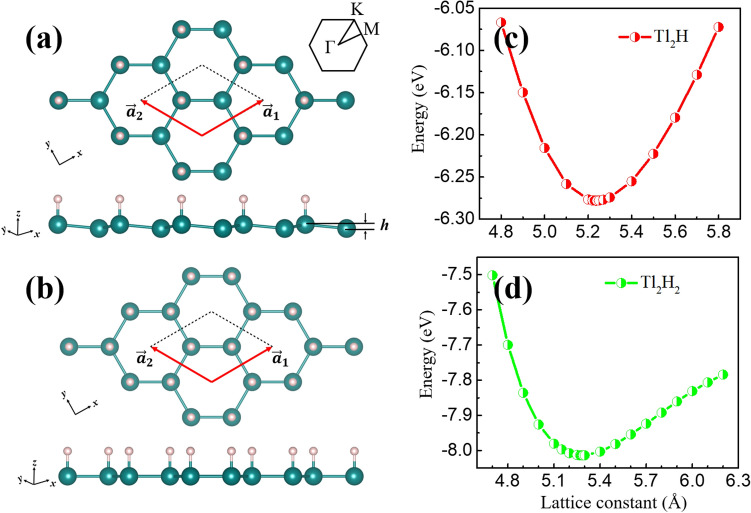
(Color online) The geometry structures of the monolayer (**a**) Tl_2_H and (**b**) Tl_2_H_2_ from the top and side views. The buckled height between the Tl atoms is *h*. The inset shows the first BZ with the high symmetry points. The green and white balls represent Tl and H atoms, respectively. In (**a**) and (**b**), the corresponding *xyz* axis are shown. (**c**, **d**) The total energies as a function of the lattice constants for the monolayer Tl_2_H and Tl_2_H_2_, respectively.

Different from other heavy element monolayers, the thallene monolayer is a fully flat 2D honeycomb lattice without buckling^18,19^. In other words, the Tl atoms in thallene are aligned in the same plane, with two Tl atoms in each unit cell. The structure has a space group symmetry of *P6/mmm* (No. 191) with a space inversion symmetry. On this basis, we construct two types of functionalized thallene structures to explore the unique electronic structures: unilateral semi-saturation and unilateral full saturation with hydrogen atoms, which were named as Tl_2_H and Tl_2_H_2_, respectively, as shown in Fig. 1a,b. To simulate the experimental configuration of thallene grown on a substrate^19^, merely the unilateral saturation is considered. Due to the unilateral hydrogenation, structural inversion symmetry of the two materials is broken and the space groups of Tl_2_H and Tl_2_H_2_ are *P3m1* (No.156) and *P6mm* (No.183), respectively. After hydrogenation, the Tl atoms still own a honeycomb lattice (see the top views of Fig. 1a,b) for Tl_2_H and Tl_2_H_2_, and the Tl atomic layer of monolayer Tl_2_H is not a completely planar structure, but a low-buckled structure with a vertical height *h* between the two Tl atomic layers (see the side view of Fig. 1a). In Tl_2_H_2_, the Tl atomic layer still maintains a planar structure (see the side view of Fig. 1b). The buckled structural characteristic in Tl_2_H is attributed to the different chemical environments around the two types of Tl atoms located in different positions of the honeycomb lattice. In the unit cell of Tl_2_H (Fig. 1a), the left Tl atom is not only bonded to three in-plane neighbor Tl atoms, but also saturated by one H atom. The interaction between Tl and H leads to the Tl atom (bonded to the H atom) approaching the H atom. Thus, a low-buckling structure appears for the Tl atoms in Tl_2_H (Fig. 1a). Dissimilarly, in Tl_2_H_2_, both of the Tl atoms in the unit cell bond with three neighbor Tl atoms and one H atom, resulting in a planar structure of the Tl atoms in the monolayer (Fig. 1b).

The optimized lattice constants of the Tl_2_H and Tl_2_H_2_ are *a* = 5.24 Å and 5.28 Å (Fig. 1c,d), respectively, which together with other structural parameters, the binding energies, and the formation energies, are listed in Table 1. The positions of the atoms in the unit cells for the two monolayers are given in the Supplementary Material as Table SI. Compared to the Tl–Tl bond length (${d}_{Tl-Tl}$) in pristine thallene (3.01 Å^18^ and 3.03 Å^19^), the Tl-Tl bond lengths in Tl_2_H (3.04 Å) and Tl_2_H_2_ (3.05 Å) are increased slightly by the hydrogenation. The trend is reasonable because the repulsive force between the H–H and Tl–H ions appears in the monolayer with H adsorbed. And it becomes larger with the increase of the H concentration. The binding energies and formation energies of the two monolayers are also calculated to investigate their structural stability. The binding energies are calculated by ${E}_{b/{Tl}_{2}H}=\left(2{E}_{Tl}+{E}_{H}\right)-{E}_{{Tl}_{2}H}$ and ${E}_{b/{Tl}_{2}{H}_{2}}=2\left({E}_{Tl}+{E}_{H}\right)-{E}_{{Tl}_{2}{H}_{2}}$, where ${E}_{{Tl}_{2}H}$/${E}_{{Tl}_{2}{H}_{2}}$ is the total energy per unit cell of the Tl_2_H /Tl_2_H_2_ monolayer and ${E}_{Tl}$/${E}_{H}$ is the total energy of one Tl/H atom. The positive and relatively large values (> 4.5 eV) of the obtained ${E}_{b}$ for Tl_2_H and Tl_2_H_2_ indicate very strong bonding between the atoms. The formation energies for the two monolayers are calculated with ${E}_{f/{Tl}_{2}H}={E}_{{Tl}_{2}H}-{E}_{Tl}{\prime}-1/2{E}_{{H}_{2}}$ and ${E}_{f/{Tl}_{2}{H}_{2}}={E}_{{Tl}_{2}{H}_{2}}-{E}_{Tl}{\prime}-{E}_{{H}_{2}}$, where ${E}_{Tl}{\prime}$ is the total energy per unit cell of the pristine thallene monolayer and ${E}_{{H}_{2}}$ is the total energy of one H_2_ molecule. The obtained negative formation energies for the monolayers (Table 1) show exothermic reactions from the thallene monolayer and H_2_ molecules, guaranteeing the feasibility of the experimental synthesis for these functionalized materials.

To explore the dynamical stability of the two monolayers, we calculate the phonon spectra for them. The results are displayed in Fig. S1(a) and (b). There are some negative frequencies in phonon spectra for the both Tl_2_H and Tl_2_H_2_ monolayers. The results are not surprising because the phonon spectrum of the pristine thallene has also negative frequencies, as reported in Ref. 18. To eliminate the negative frequencies, the two monolayers are deposited to SiC substrates. The geometries of the Tl_2_H_2_/SiC heterostructure are displayed in Fig. S2 (a) and (b). The substrates are helpful to stabilize the dynamical stability for the two materials. There are, however, still some negative frequencies for the Tl_2_H/SiC heterostructure, with the lowest value of − 0.7 THz (larger than that of the material without the substrate, − 2.0 THz). All the soft modes in the Tl_2_H_2_ monolayer are removed by the SiC substrate, as indicated in Fig. S2(c). Thus, to experimentally observe the interesting electronic states and topological behaviors in the materials, the two monolayers should be placed on the SiC substrates. There are rich examples that the materials are not completely dynamically stable (with some soft vibration modes), which could be, however, fabricated successfully in experiments, for such as stanene^21,22^ and plumbene^23,24^.

## Band structures and strain tuning

**Figure 2:**
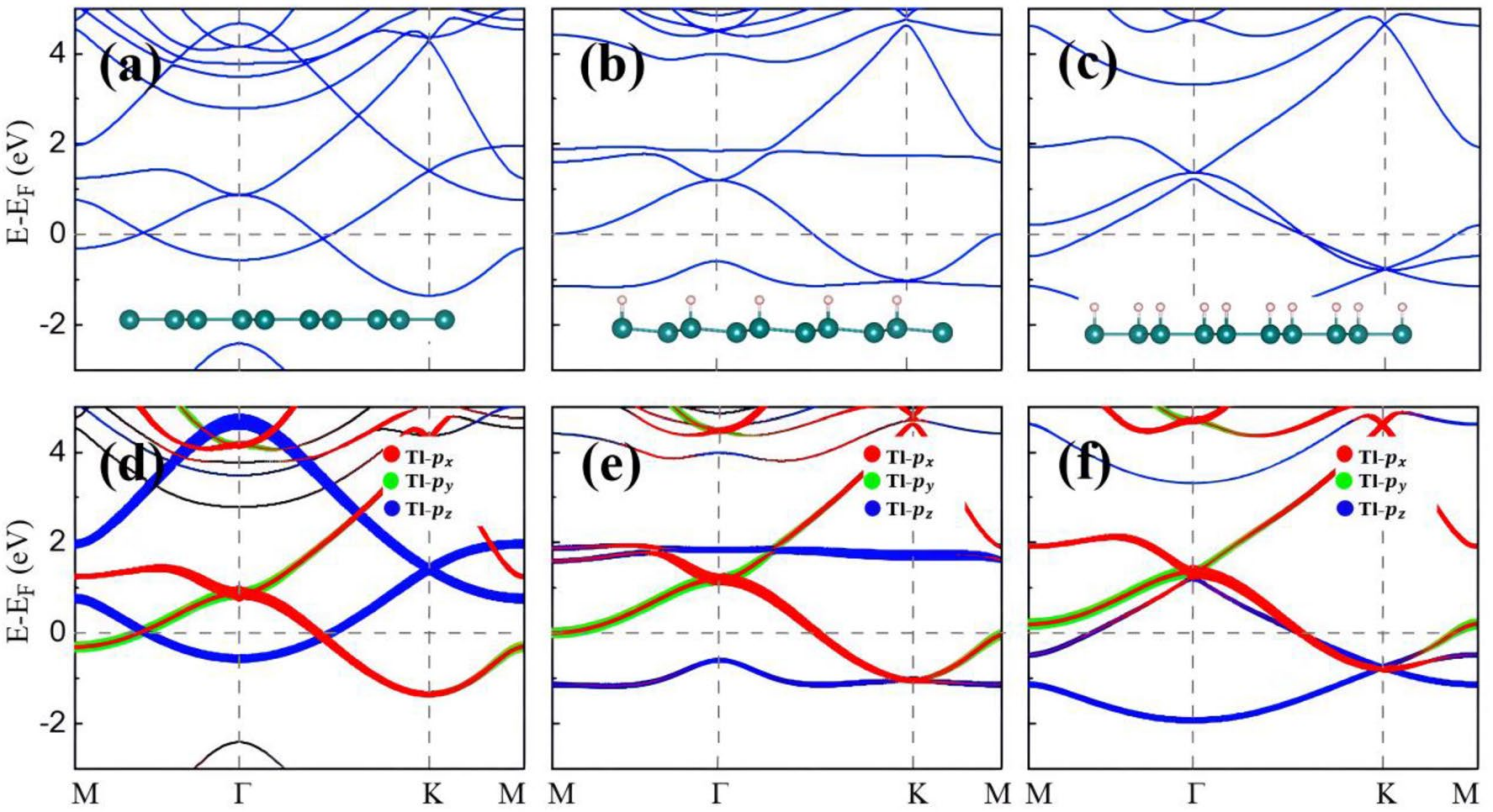
Band structures for (**a**) Tl, (**b**) Tl_2_H, and (**c**) Tl_2_H_2_ monolayers without SOC. (**d**–**f**) are orbital projections corresponding to (**a**–**c**) band structures, respectively. The insets in (**a**–**c**) are the side views of the corresponding structures.

Figure 2 shows band structures of the hydrogenated Tl_2_H and Tl_2_H_2_ monolayers without the consideration of SOC. For convenient comparison, the bands of the pristine 2D thallene are also displayed and discussed first. Similar to the case of the low-buckled plumbene^17^, a linear Dirac cone composed of Tl *p*_*z*_ orbitals is found around the K point (at about 1.4 eV in Fig. 2a,d). Due to the *C*_*3v*_ symmetry of the honeycomb structure, another set of twofold degenerate bands appear at the Γ point around 1.0 eV in the planar thallene, composed of Tl *p*_*x*_ and* p*_*y*_ orbitals (Fig. 2a,d). The dispersion of these *p*_*x*_ and* p*_*y*_ orbitals belongs to quadratic non-Dirac bands since the Γ point is a time-reversal invariant point and the linear *k* term in the energy eigenvalue at such point is forbidden^25^. This band feature with two twofold degenerate points respectively at Γ and K also presents in Pb monolayers^17^, which happen at the Fermi level (E_F_). They are, however, located above the E_F_ in the Tl monolayer due to the less valence electron number of a Tl atom, compared to a Pb atom. In Tl_2_H, since partial Tl *p*_*z*_ orbitals are saturated by hydrogen atoms, the linear Dirac degenerate bands around the K point are opened and become two relatively flat bands (the blue curves in Fig. 2e). The tendency is the same as those in half-hydrogenated Bi honeycomb monolayers in nonmagnetic states^26^. The flat bands of the Tl *p*_*z*_ orbitals in Tl_2_H are, however, not located around the E_F_. Thus, spontaneous spin polarization does not happen in Tl_2_H, different from many other half-hydrogenated Ge^27^, Sn^27^, and Bi^26^ monolayers etc. The quadratic non-Dirac bands (composed of Tl *p*_*x*_ and* p*_*y*_ orbitals) at the Γ point still exist because of the *C*_*3v*_ symmetry unbroken by the hydrogenation. For the electronic structure of Tl_2_H_2_ (Fig. 2c,f), it can be intuitively understood that the dangling bonds of Tl *p*_*z*_ are all saturated by H atoms now, which makes the upper flat *p*_*z*_ orbitals move down in energy.

**Figure 3:**
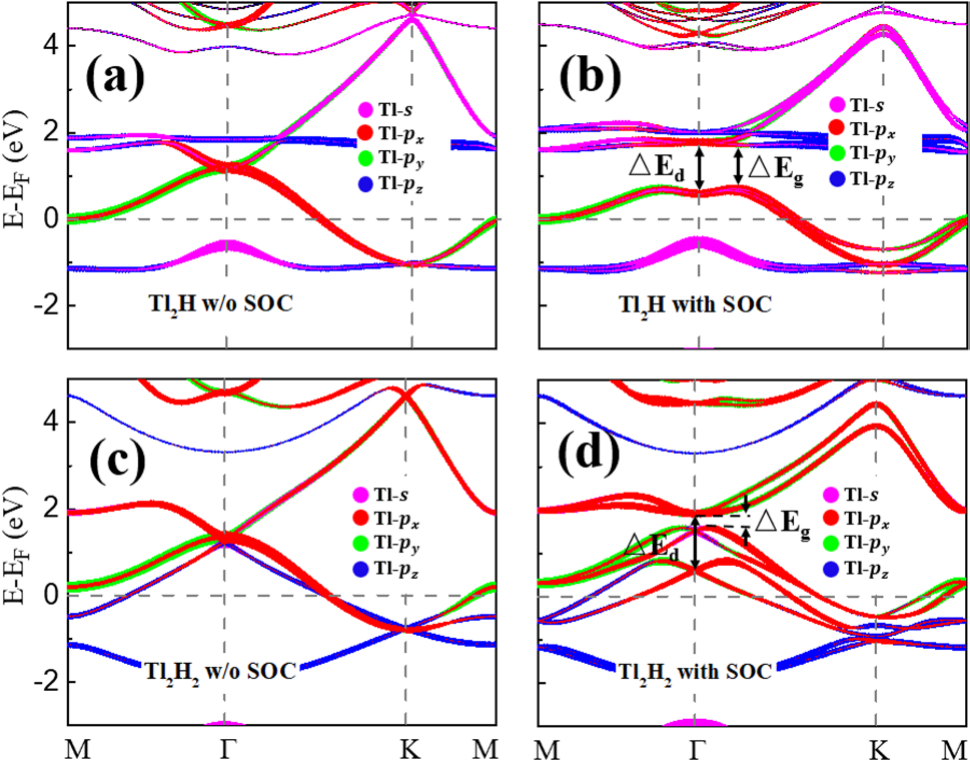
(**a**) and (**b**) give the orbital-projected band structures of the Tl_2_H without and with SOC, respectively. (**c**) and (**d**) give the orbital-projected band structures of the Tl_2_H_2_ without and with SOC, respectively. The dot size is proportional to the contribution of the corresponding orbitals. In (**b**) and (**d**), ΔE_d_ represents a direct band gap at the Γ point and ΔE_g_ represents the global band gap.

The orbital-resolved band structures with SOC interactions for the Tl_2_H and Tl_2_H_2_ monolayers are shown in Fig. 3. As displayed in Fig. 3a,b, the bands around the E_F_ in Tl_2_H are primarily made up of the Tl *p* orbitals (and also some *s* orbitals below the E_F_). When the SOC interaction is taken into account, the twofold degeneracy at the Γ point is lifted and a direct band gap ΔE_d_ = 1.19 eV is opened at the Γ point (Fig. 3(b)). This large band gap can be ascribed to the strong on-site SOC interaction contributed by Tl *p*_*x*_ and* p*_*y*_ orbitals^28^. Due to the band dispersion, a relatively small global band gap ΔE_g_ = 854 meV is obtained for Tl_2_H, which is pretty large and close to the record value of QSH effects reported in the F-decorated Bi monolayer^29^. For Tl_2_H_2_, the quadratic non-Dirac degenerate point (red and green colors) is located at 1.3 eV at the Γ point (Fig. 3c), below which there is an energy band primarily composed of the Tl *p*_*z*_ orbitals (blue color). Under the influence of SOC, not only the quadratic non-Dirac point is broken with a band gap opened, but also the band inversion between Tl *p*_*x/y*_ and Tl *p*_*z*_ orbitals is induced in Tl_2_H_2_. Due to the large on-site SOC, the SOC-induced direct band gap of the Tl *p*_*x/y*_ is still very large (ΔE_d_ = 1.32 eV, as marked in Fig. 3d), within which there is the band of the Tl *p*_*z*_ orbitals hybridizing with Tl *p*_*x/y*_ orbitals. Thus, the band inversion between Tl *p*_*x/y*_ and Tl *p*_*z*_ orbitals causes the global band gap (334 meV) for the monolayer Tl_2_H_2_ much less than that (854 meV) of Tl_2_H. These SOC-induced global band gaps in the two monolayers are expected to be topologically nontrivial^30^, to be discussed below. Compared to the pristine thallene with a topologically trivial band gap (98 meV)^18^, the hydrogenation causes the thallene becoming a metal. Especially, for the full passivated thallene, more bands cross the E_F_ and the Tl_2_H_2_ monolayer turns to be a typical metal. This trend is opposite to the full-passivation effect in group IVA monolayer. For example, the full-hydrogenation makes graphene change from a gapless semiconductor to a semiconductor with a large band gap of 3.49 eV^31^ due to the Dirac cone composed of C *p*_*z*_ orbitals located right at E_F_ in the pristine graphene.

**Figure 4:**
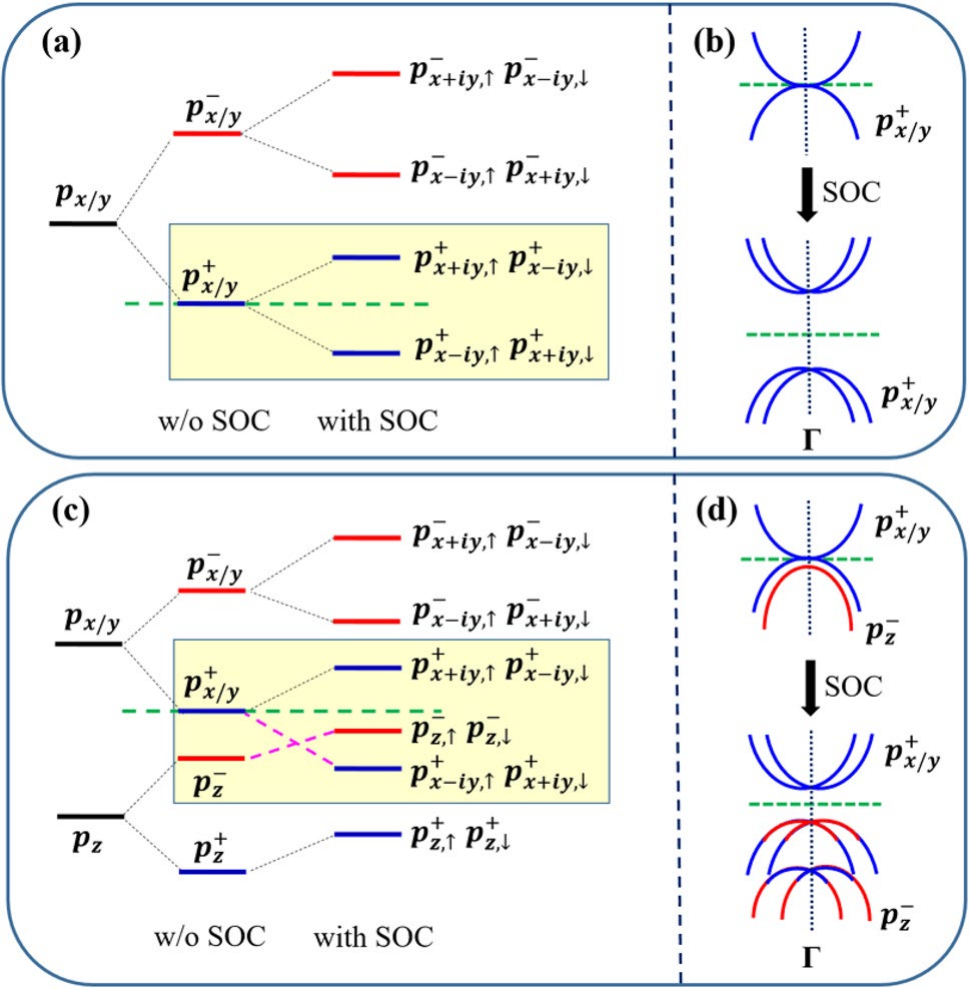
(Color online) (**a**) and (**c**) correspond to the Tl-orbital evolution diagrams at Γ point near the E_F_ in the monolayer Tl_2_H and Tl_2_H_2_, respectively. The signs of ' + ' and '−' indicate the bonding and anti-bonding states, respectively. The green dotted lines represent the location of the concerned energy degenerate points. (**b**) and (**d**) show the schematic band dispersion of the energy regions marked by the rectangles in (**a**) and (**c**), respectively. In (**b**) and (**d**), the blue curves represent the bonding states of the *p*_*x/y*_ orbitals and the red curves represent the anti-bonding states of the *p*_*z*_ orbital.

Since the distributions of the featured bands for the Tl_2_H and Tl_2_H_2_ monolayers are different, the mechanisms of the band evolution for the two materials are now analyzed. For Tl_2_H and Tl_2_H_2_ monolayers, the evolutions of the atomic orbitals around the Γ point are both mainly triggered by two aspects: one is chemical bonding and the other is SOC. The difference is that the two Tl *p*_*z*_ bands in the monolayer Tl_2_H are far away from the concerned energy degenerate point at Γ (Fig. 2e). Since we mainly focus on the bands near the two-fold degenerate point, only the Tl *p*_*x/y*_ orbitals are discussed in Tl_2_H. As shown in Fig. 4a, in the absence of SOC, the chemical bonding between Tl-Tl atoms forms bonding and anti-bonding states for the *p*_*x/y*_ orbitals. These states are labeled as $|{p}_{x/y}^{\pm }\rangle$, where the superscript (+ /$-$) denotes the bonding/anti-bonding states and the concerned degenerate energy level is mainly contributed by the ${p}_{x/y}^{+}$ orbitals. When SOC is turned on, $|{p}_{x/y}^{+}\rangle$ splits into two energy levels. Analogous to the cases of stanene^15^ and germanene films^16^, the two SOC-induced splitting energy levels can be expressed as $\left|{p}_{x+iy,\uparrow }^{+}\right.,{p}_{x-iy,\downarrow }^{+}\rangle$ and $\left|{p}_{x-iy,\uparrow }^{+}\right.,{p}_{x+iy,\downarrow }^{+}\rangle$ (Fig. 4a), with $\left|{p}_{x+iy,\uparrow }^{+}\right.,{p}_{x-iy,\downarrow }^{+}\rangle$ moving up and the other set of states moving down. Thus, a band gap is opened at the Γ point. Note that the role of SOC here is only to lift the degeneracy or open a band gap. It does not induce band inversion, as displayed in Fig. 4b. The status in Tl_2_H_2_ is, however, dissimilar to that of Tl_2_H. The effect of the Tl *p*_*z*_ orbital cannot be ignored in Tl_2_H_2_ due to some of its locations close to the two-fold degenerate point at the Г point (Fig. 2f). As illustrated in Fig. 4c, the chemical bonding between Tl-Tl atoms makes all atomic orbitals split into bonding and anti-bonding states, labeled as $|{p}_{x/y}^{\pm }\rangle$ and $|{p}_{z}^{\pm }\rangle$, respectively. Owing to the increase of the H concentration in Tl_2_H_2_, more $|{p}_{z}^{-}\rangle$ states are passivated. Hence, the $|{p}_{z}^{-}\rangle$ state moves down in energy and is lower than the two-fold degenerate $|{p}_{x/y}^{+}\rangle$ states (Fig. 4c). When the SOC is taken into account, $|{p}_{x/y}^{+}\rangle$ splitting is similar to that of Tl_2_H, where $\left|{p}_{x+iy,\uparrow }^{+}\right.,{p}_{x-iy,\downarrow }^{+}\rangle$ moves down and the anti-bonding state of the *p*_*z*_ orbitals (labeled as $\left|{p}_{z,\uparrow }^{-}\right.,{p}_{z,\downarrow }^{-}\rangle$) moves up. The large SOC splitting of the $|{p}_{x/y}^{+}\rangle$ states leads to the band inversion between *p*_*x/y*_ and *p*_*z*_ states (Fig. 4d), resulting in a decrease of the band gap in Tl_2_H_2_ (Fig. 4d), compared to that of Tl_2_H. To confirm the results, the electronic structures of the two monolayers are also calculated with the hybrid density functional (HSE06). Similar band structures with the indirect band gaps of 924 and 233 meV are obtained for Tl_2_H and Tl_2_H_2_, respectively (Fig. S3).

**Figure 5:**
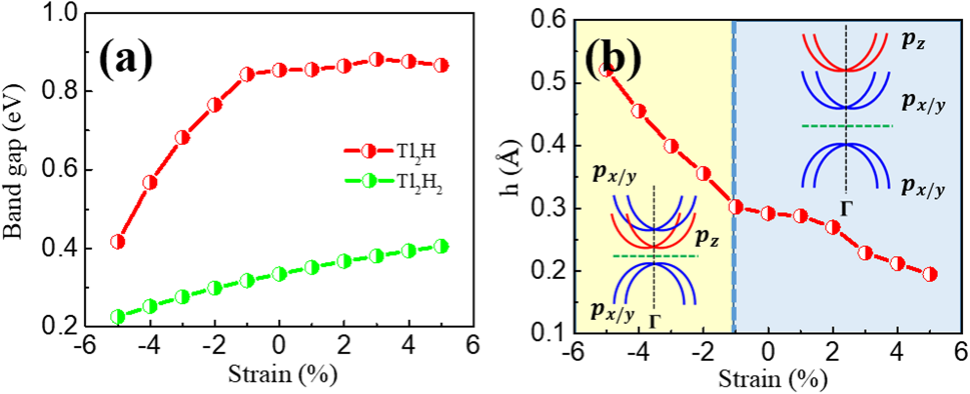
(Color online) (**a**) The global band gaps as a function of the strain for the monolayer Tl_2_H and Tl_2_H_2_. The red and green colors give the Tl_2_H and Tl_2_H_2_ results, respectively. (**b**) The buckled heights (*h*) between Tl atoms in the monolayer Tl_2_H as a function of the strain. The inset shows the band evolution, in which the blue/red colors express the *p*_*x/y*_/* p*_*z*_ bands.

Applying strain is generally an effective strategy for making controls to the structures and electronic states of the monolayer materials. Here, we apply biaxial strain in the range of − 5 to 5% to the two monolayers. To check the structural stability of the monolayers under strain, the binding energies of the two structures under strain are calculated. As illustrated in Fig. S4, the strain does not decrease much the binding energies, indicating the structural stability of the monolayers under strain. The trend is consistent with the results reported in Ref.^32^ that thallene can undergo very large tensile strain (~ 27%) in experiments. The band gaps opened at the two-fold degenerate point of the non-Dirac bands as a function of the biaxial strain for the monolayers are shown in Fig. 5a. The strain is defined as (*a*′*-a*)$\times$ 100% */a*, where *a*′ stands for the lattice constant with strain applied and *a* stands for the equilibrium lattice constant. Due to the different orbital components near the concerned band gaps for the Tl_2_H and Tl_2_H_2_, the responses to the external tuning are various.

The global band gaps for Tl_2_H exhibit roughly linear and saturated trends with respect to the compressive and tensile strain, respectively (Fig. 5a), comprehended as following. As indicated in Fig. S5, with the strain varying from -3 ~ 3%, the *p*_*x/y*_ states of Tl_2_H move down in energy while the *p*_*z*_ states (at about 1.6 eV) move up. And a band inversion occurs with *p*_*x/y*_ and *p*_*z*_ orbitals around the Г point at -1% strain (Fig. S5). During this process, the buckled height (*h*) decreases (Fig. 5b), which can be straightforward ascribed to the increase of the in-plane lattice constants. The *h* decrease makes the *p*_*z*_ orbitals of the Tl atoms without H atoms more isolated. The interaction between this Tl atom and its three neighbor Tl atoms more tends to form planar *sp*^2^ hybridization instead of three dimensional *sp*^3^ hybridization, giving rise to the Tl *p*_*z*_ orbitals moving up in energy during the process (especially around Γ point). This tendency causes the band inversion between the *p*_*x/y*_ and *p*_*z*_ orbitals at − 1% strain, as illustrated in the inset of Fig. 5b. Since the SOC split of *p*_*x/y*_ orbitals does not change much with the strain (see the black arrows in Fig. S5), the band gap hardly changes with the increase of the tensile strain (Fig. 5a).

Different from the case of Tl_2_H, the band gap of Tl_2_H_2_ increases linearly with the biaxial strain varying from − 5 to 5% (Fig. 5a). The overall tendency is, however, relatively weak. The increase of the band gap for Tl_2_H_2_ can be primarily attributed to two factors. One is that the band dispersion tends to weaken with the increase of the atomic distance. The other is that the on-site SOC of *p*_*x/y*_ orbitals is enhanced slightly by the gradual decrease of the *p*_*z*_ state involved in the concerned bands with the strain varying from − 5 to 5% (Fig. S6).

### Strong Rashba effect

**Figure 6:**
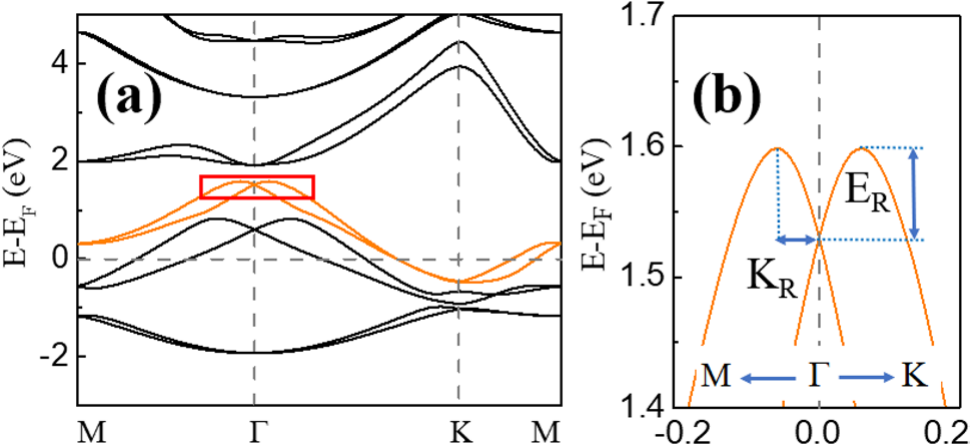
(**a**) Band structure of the Tl_2_H_2_ monolayer with SOC. (**b**) The magnified view of the band structure (in the red rectangle in (**a**)) of the Tl_2_H_2_ monolayer. The orange color indicates the concerned bands.

**Figure 7:**
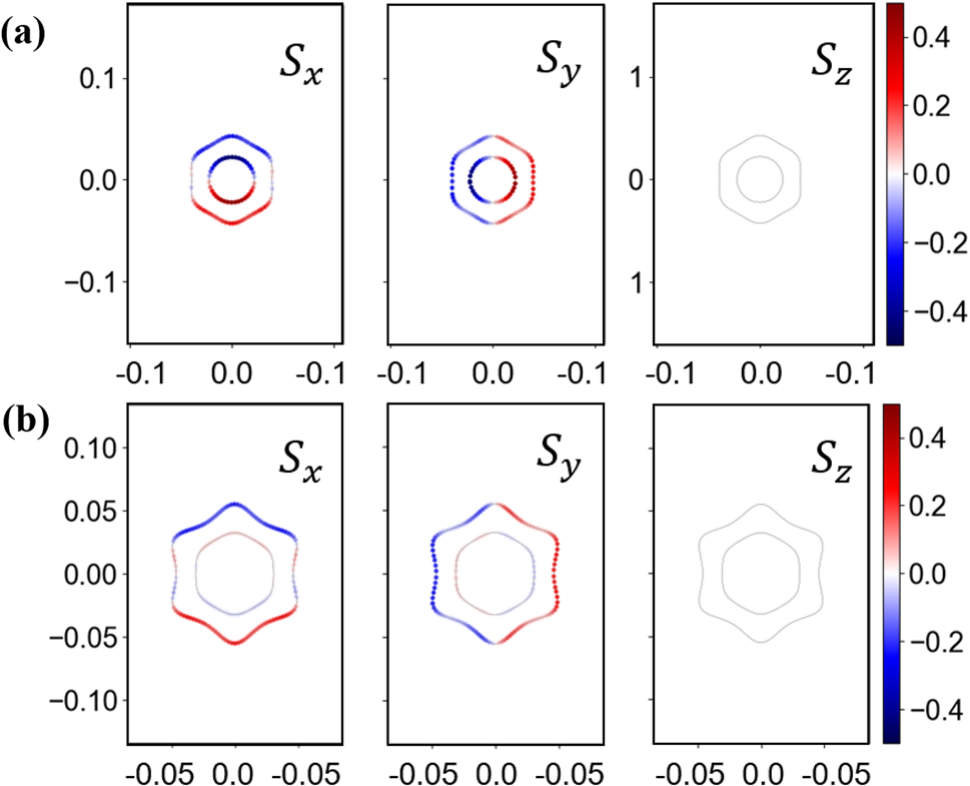
(Color online) Spin textures centered at the Г point calculated in the ${k}_{z}=0$ plane for the Tl_2_H_2_ monolayer with (**a**) the pristine structure and (**b**) 20% tensile strain, respectively. The red and blue colors show spin-up and spin-down states, respectively. The energy in (**a**) is set at 1.1 eV.

Considerable Rashba SOC interactions^32^ exist in the monolayer Tl_2_H and Tl_2_H_2_ due to the breaking of vertical reflection symmetry. The strength of the Rashba SOC generally can be manipulated markedly by an external electric field. And the Rashba SOC effect has been employed to fabricate all-electric spintronic devices, such as spin field effect transistors and spin valves (without magnetic fields needed)^33–35^, promoting the spintronic applications. Very large Rashba spin splitting has been reported for the deep-energy π states (~ − 8 eV below E_F_) of graphene deposited on metallic substrates^36^. Here, we merely discuss the Rashba effect of Tl_2_H_2_ since it is stronger than that of Tl_2_H (Fig. 3b,d). The orange bands of Tl_2_H_2_ in Fig. 6 are focused on. The magnified bands around the Г point (Fig. 6b) exhibit an obvious Rashba splitting phenomenon similar to that of semiconductor quantum wells and heavy metal surfaces^37–41^. To examine the Rashba effect, the spin textures in the ${k}_{x}$–${k}_{y}$ plane for Tl_2_H_2_ are calculated. The spin-projected constant-energy contour plots for the spin textures calculated in the ${k}_{z}=0$ plane are shown in Fig. 7a. The flower-like spin textures appear in Tl_2_H_2_. For both *S*_*x*_ and *S*_*y*_ spin components, the pair of spin-splitting bands have the same spin orientation. However, for pure 2D Rashba spin splitting, the pair of spin-splitting bands for the both *S*_*x*_ and *S*_*y*_ spin components generally have opposite spin orientations^42^. For Tl_2_H_2_, the SOC effect not only opens the nontrivial band gap, but also induces the band inversion near E_F_ (Fig. 4c,d), resulting in an anomalous spin texture. To verify this point, the *p*_*z*_ state is moved down in energy to the position far from the concerned energy point by applying 20% biaxial tensile strain. In this case, the SOC is not sufficient to reverse the bands and the corresponding spin structures of the Tl_2_H_2_ monolayer are displayed in Fig. 7b. Clearly, the pair of spin-splitting bands for both *S*_*x*_ and *S*_*y*_ spin components have opposite spin orientations. Due to the disappearance of the *S*_*z*_ component, the spin moments of the two rings shown in Fig. 7b have opposite chirality. The large ring is anticlockwise, while the small ring is clockwise. This large Rashba effect has recently been observed in experiments in the thallene^20^ with a different forming mechanism.

**Figure 8:**
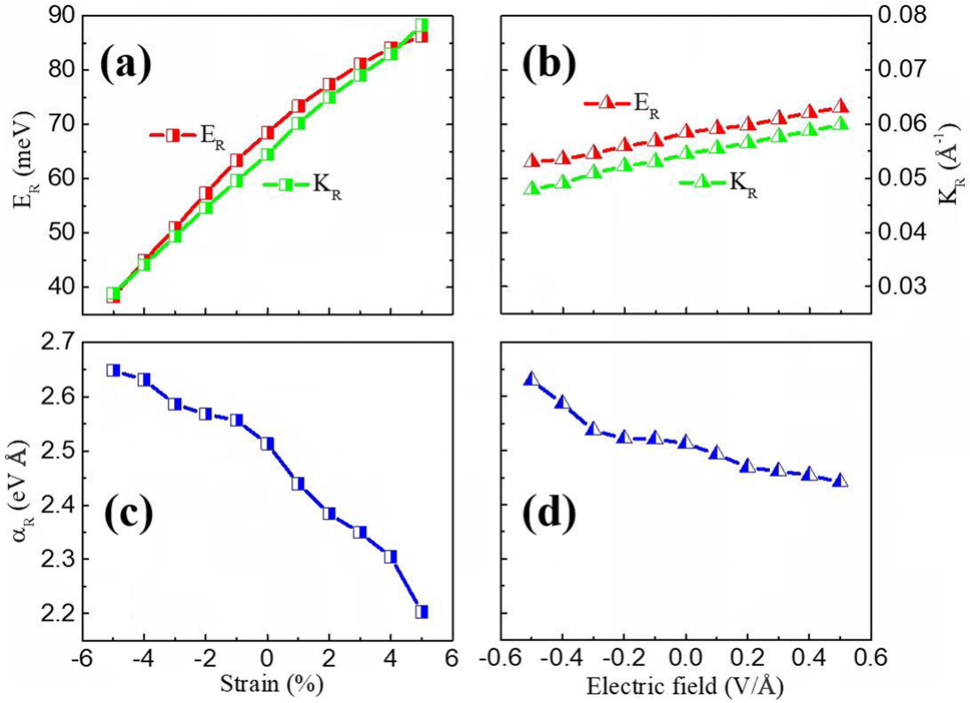
The Rashba energy E_R_ (red color) and the momentum offset K_R_ (green color) under biaxial strain (**a**) and external electric fields (**b**) in the monolayer Tl_2_H_2_. The Rashba parameter α_R_ under different biaxial strain (**c**) and external electric fields (**d**) is also shown.

The Rashba coefficient α_R_ is also calculated to describe the strength of the Rashba SOC effect. The Rashba coefficient can be obtained from the formula α_R_ = 2E_R_/K_R_^41^, where E_R_ and K_R_ are defined in Fig. 6b. From Fig. 6b, the E_R_, K_R_, and α_R_ are estimated to be 68 meV, 0.054 Å^−1^, and 2.52 eVÅ, respectively. The obtained α_R_ is significantly higher than those of many heterostructures or surface states of 2D TIs, such as InGaAs/InAlAs (0.07 eVÅ)^34^, Au/W(110) (0.16 eVÅ)^35^, and CSb_3_ (0.83 eVÅ)^43^. In Fig. 8, we show the Rashba energy E_R_, the momentum offset K_R_, and Rashba parameter α_R_ under different biaxial strain (− 5 to 5%) and external electric fields (− 0.5 to 0.5 V/Å). With the increase of the biaxial strain and external electric field, the values of E_R_ and K_R_ increase almost linearly and are very sensitive to the biaxial strain. On the contrary, the values of α_R_ shows a decreasing trend with the increase of the biaxial strain and external electric field, indicating that the application of compressive strain and electric fields along $-z$ axis are more conducive to the Rashba effect of Tl_2_H_2_. Under compressive strain, the Tl *p*_*z*_ component of the concerned bands becomes more (Fig. S6), which together with the slight increase of Tl-H bond strength (the bond length becomes short) and the Tl *p*_*x/y*_ asymmetric distribution about the Tl-Tl plane results in the enhancement of the Rashba effect with the compressive strain in Tl_2_H_2_. As shown in Fig. 1b, the hydrogen atoms are located above the Tl atoms. When a negative electric field (along $-z$ axis) is applied, the electrons of thallene tend to move toward the H atoms. And the charge densities between the Tl-H atoms become more, leading to the increase of the Rashba effect.

### Large band-gap quantum spin Hall state

**Figure 9:**
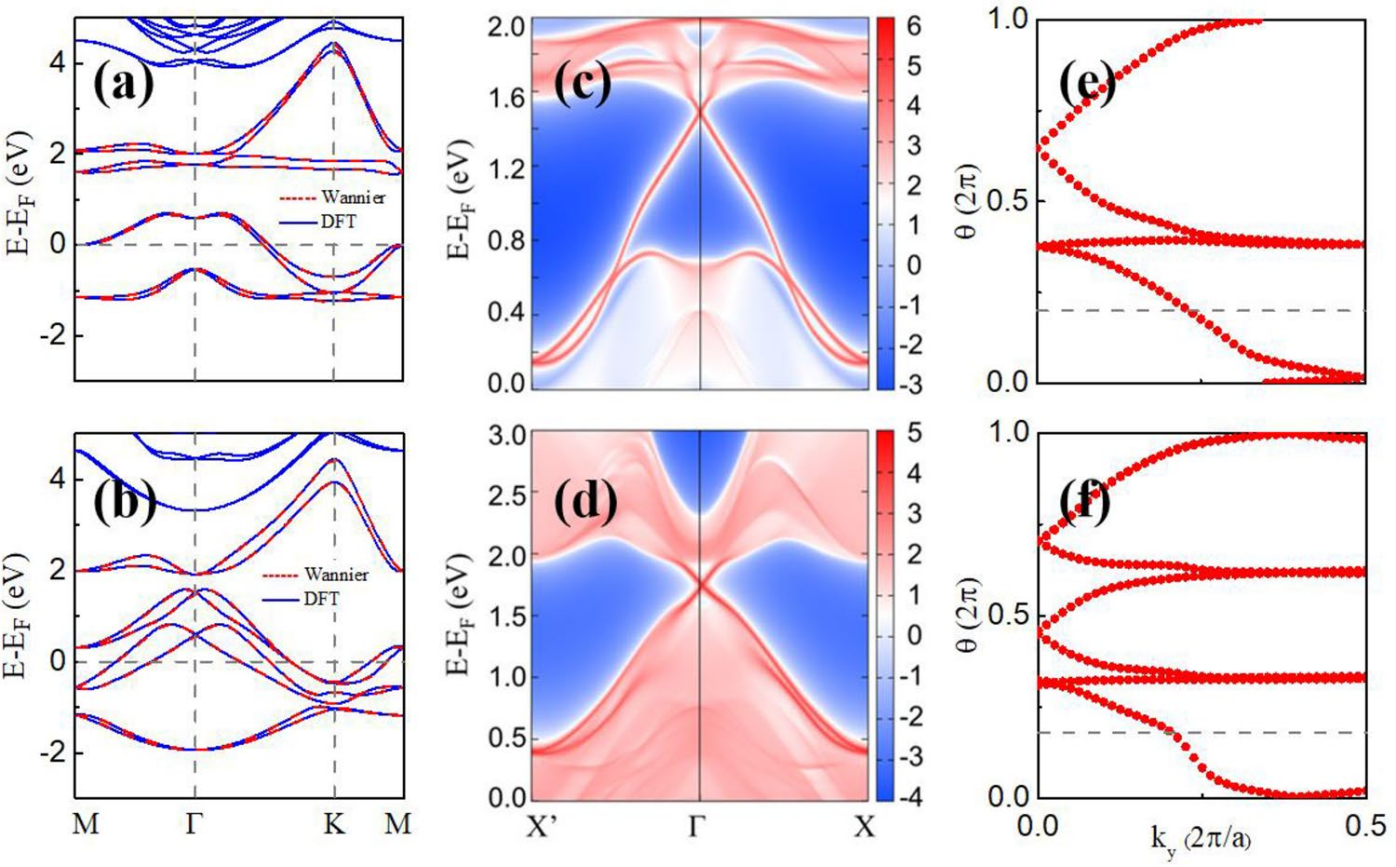
Band structures for the (**a**) Tl_2_H and (**b**) Tl_2_H_2_ monolayers with SOC by using Wannier interpolation (red dotted curves) calculations. The DFT results (blue solid curves) are also shown. (**c**, **d**) The calculated edge states for the Tl_2_H and Tl_2_H_2_ monolayers. Evolutions of the Wannier function centers for Tl_2_H (**e**) and Tl_2_H_2_ (**f**) along k_y_, yielding Z_2_ = 1.

The topological properties of the concerned band gaps for the monolayer Tl_2_H and Tl_2_H_2_ are investigated. Figure 9a,b show the band structures of the monolayer Tl_2_H and Tl_2_H_2_ with SOC. The acquired bands from the Wannier interpolation method are also displayed, which are in very good agreement with those obtained from the DFT calculations. Figure 9c,d give the edge states of the semi-infinite Tl_2_H and Tl_2_H_2_ samples by using the Green’s function method and the semi-infinite MLWF Hamiltonian. Obviously, the topological protected gapless helical edge states connect the bands at the two sides of the band gaps, as expected. To further identify the topological properties of the gapped states of the monolayer Tl_2_H and Tl_2_H_2_, the topological invariant Z_2_ is obtained by using the Wannier charge center (WCC) method^44^. The calculated Z_2_ = 1 for the monolayer Tl_2_H and Tl_2_H_2_ with the ${\mathrm{E}}_{\mathrm{F}}$ set to be located inside the band gaps indicates that the gaped states are QSH states with topological nontrivial band gaps induced by SOC (Fig. 9e–f). The E_F_ movement can be achieved experimentally through such as carrier doping with the carrier concentration of 4.1 $\times$ 10^14^ and 4.2 $\times$ 10^14^ cm^−2^ for Tl_2_H and Tl_2_H_2_, respectively, which can be realized in experiments via current advanced gating technologies^45^. Our research results indicate that the QSH states with large band gaps can be achieved in the thallene film by functionalization. No strong tensile strain is needed to acquire the QSH state in Tl_2_H and Tl_2_H_2_, superior to the pristine thallene^18^. The obtained QSH states are actually very robust against the strain. As indicated in Fig. 5a, the global band gaps for Tl_2_H and Tl_2_H_2_ are both not closed under the strain varying from − 5 to 5%. Thus, the QSH states are well maintained even under 5% tensile or compressive strain.

**Figure 10:**
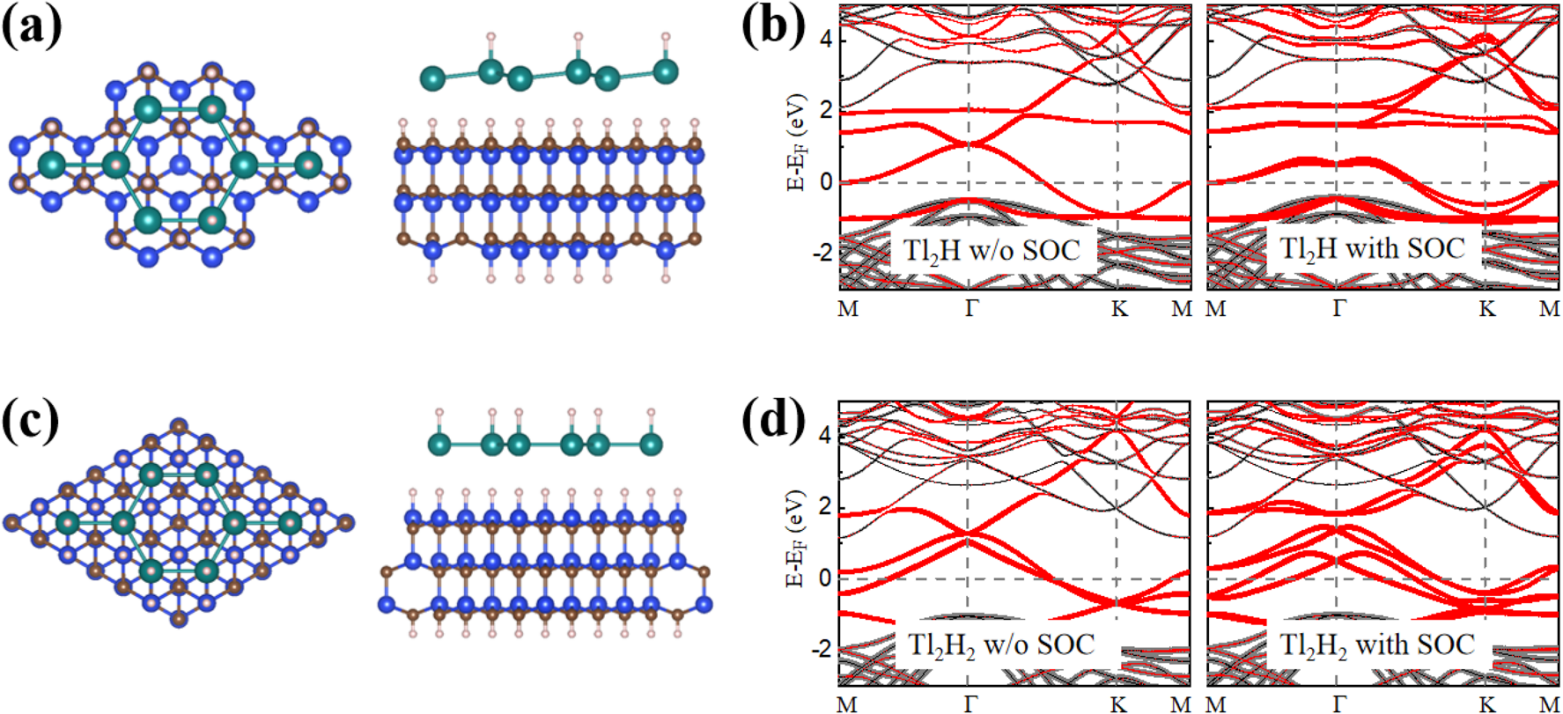
The geometry structures of the (**a**) Tl_2_H and (**c**) Tl_2_H_2_ monolayers placed on a threefold symmetric SiC (0001) substrate from the top and side views, respectively. The substrate is modeled as a SiC three-layer, saturated with H atoms in the bottom and top surfaces. The unit cell of the system is in a commensurate ($\sqrt{3}\times \sqrt{3}$) reconstruction of SiC (0001). (**b**) and (**d**) give the band structures of the Tl_2_H and Tl_2_H_2_ monolayers on the SiC (0001) substrate without and with SOC, respectively.

To check the accessibility of the topological states for Tl_2_H and Tl_2_H_2_ in experiments, we deposit Tl_2_H and Tl_2_H_2_ monolayers to a substrate material of SiC (0001). The built geometrical structures of the Tl_2_H/SiC and Tl_2_H_2_/SiC heterostructures are displayed in Fig. 10a,c. Note that the bottom and top surfaces of the SiC substrate are saturated with H atoms to remove the dangling bonds of the surface atoms for the SiC substrates. The lattice mismatch between the Tl_2_H /Tl_2_H_2_ sample and the SiC (0001) substrate is very small (about 1.82%/0.96%). The optimized interface distance between the sample and the substrate is 2.32 Å/2.77 Å, indicating the van der Waals (vdW) interactions in the interface. The achieved band structures are shown in Fig. 10b,d. The bands near the E_F_ of Tl_2_H and Tl_2_H_2_ are obviously not affected much by the SiC substrate, due to the weak vdW interactions from the substrates. Therefore, the QSH states displayed in Fig. 9a,b keep well in the samples when depositing on the SiC substrate. The acquired SiC substrate together with the large binding energies (> 4.5 eV) and the negative formation energies for Tl_2_H and Tl_2_H_2_ indicates the experimental accessibility of the unique electronic and topological states in the two materials.

## Conclusions

In summary, we have built two hydrogenated thallene Tl_2_H and Tl_2_H_2_ monolayers and explored systematically their electric structures and topological properties based on density functional and crystal field theories. Our results indicate that non-Dirac quantum spin Hall states with large band gaps can be achieved in thallene by unilateral hydrogenation. The topological nontrivial band gap can be up to 855 meV, much higher than that of most of the proposed quantum spin Hall insulator. The QSH states in both monolayers are very robust and well maintained even under 5% tensile or compressive strain. Due to the absence of structural inversion symmetry, the Tl_2_H_2_ monolayer exhibits very strong Rashba spin splitting characteristics. The non-Dirac quantum spin Hall state and Rashba effect can be tuned efficiently by applying biaxial strain and external electric fields. Our results provide an excellent material platform for realizing room-temperature topological electronic devices.

## Models and methods

The geometry optimization and electronic structure calculations of the functionalized thallene are performed based on density functional theory (DFT) with the projector augmented wave method as implemented in the Vienna ab initio simulation package (VASP)^46^. The Perdew-Burke-Ernzerhof generalized gradient approximation (GGA-PBE) is adopted for the exchange–correlation functional^47^. The cutoff energy is set as 550 eV for the plane-wave basis and the vacuum space along the *c* axis is set to about 15 Å to avoid the interactions between the two adjacent slabs. The first Brillouin zone (BZ) is sampled with *k* meshes of $15\times 15\times 1$ by using Gamma-centered Monkhorst–Pack method^48^. The convergence threshold for the total energy is set to 1 × 10^−6^ eV. All the atoms are allowed to relax until the force on each atom is less than 0.01 eV/Å, which is calculated according to the Hellmann–Feynman theorem. The structural optimization is performed without the symmetry constraint. The maximally localized Wannier functions (MLWFs) are constructed by employing the WANNIER90 code^49^, in which the edge states are calculated with an iterative Green function method^50,51^. In addition, the screened exchange hybrid density functional by Heyd–Scuseria–Ernzerhof (HSE06)^52^ is also adopted for confirming the electronic structures.

**Table 1:** The calculated equilibrium lattice constants (*a*), Tl − Tl bond lengths (${d}_{Tl-Tl}$), Tl − H bond lengths (${d}_{Tl-H}$), vertical distances between the two Tl atomic planes (*h*), the binding energies (${E}_{b}$), the formation energies (${E}_{f}$), and the global band gaps (ΔE_g_) for the monolayer Tl_2_H and Tl_2_H_2_.

	*a* (Å)	*d*_Tl-Tl_ (Å)	*d*_Tl-H_ (Å)	*h* (Å)	*E*_*b*_ (eV)	*E*_*f*_ (eV)	ΔE_g_ (meV)
Tl_2_H	5.24	3.04	1.86	0.29	4.57	− 1.62	854
Tl_2_H_2_	5.28	3.05	1.87	0	5.18	− 2.23	334

### Supplementary Information

Supplementary Information.

## Data Availability

The datasets used and/or analysed during the current study available from the corresponding author on reasonable request.
