# Supplementary material for: Large-band-gap non-Dirac quantum spin Hall states and strong Rashba effect in functionalized thallene films
Source: Sci Rep. 2023 Sep 25;13:15966. doi: 10.1038/s41598-023-43314-4 (PMC10519994; doi:10.1038/s41598-023-43314-4)
Supplement: Supplementary file 1 — Supplementary Information. [file 41598_2023_43314_MOESM1_ESM.docx]

**Large-band-gap non-Dirac quantum spin Hall states and strong Rashba effect in functionalized thallene films**

Xiaojuan Liu^1^, Zhijian Li^1^, Hairui Bao^1^, and Zhongqin Yang^1,2^*

*^1^State Key Laboratory of Surface Physics, Key Laboratory of Computational Physical Sciences (MOE) and Department of Physics, Fudan University, Shanghai 200433, China*

*^2^Shanghai Qi Zhi Institute, Shanghai 200030, China*

**Email:* [*zyang@fudan.edu.cn*](mailto:zyang@fudan.edu.cn)

**TABLE SI.** The calculated equilibrium lattice constants (*a* and *c*) and atom positions for the Tl_2_H and Tl_2_H_2_ monolayers.

|  | Lattice constants  (Å) | Atoms | Position | | |
| --- | --- | --- | --- | --- | --- |
|  |  |  | x | y | z |
| Tl_2_H | *a* = 5.24 | Tl_1_ | 0.3333 | 0.6667 | 0.5068 |
|  | *c* = 19.71 | Tl_2_ | 0.6667 | 0.3333 | 0.4920 |
|  |  | H | 0.3333 | 0.6667 | 0.6012 |
| Tl_2_H_2_ | *a* = 5.28 | Tl_1_ | 0.3333 | 0.6667 | 0.5015 |
|  | *c* = 19.33 | Tl_2_ | 0.6667 | 0.3333 | 0.5015 |
|  |  | H_1_ | 0.3333 | 0.6667 | 0.5985 |
|  |  | H_2_ | 0.6667 | 0.3333 | 0.5985 |


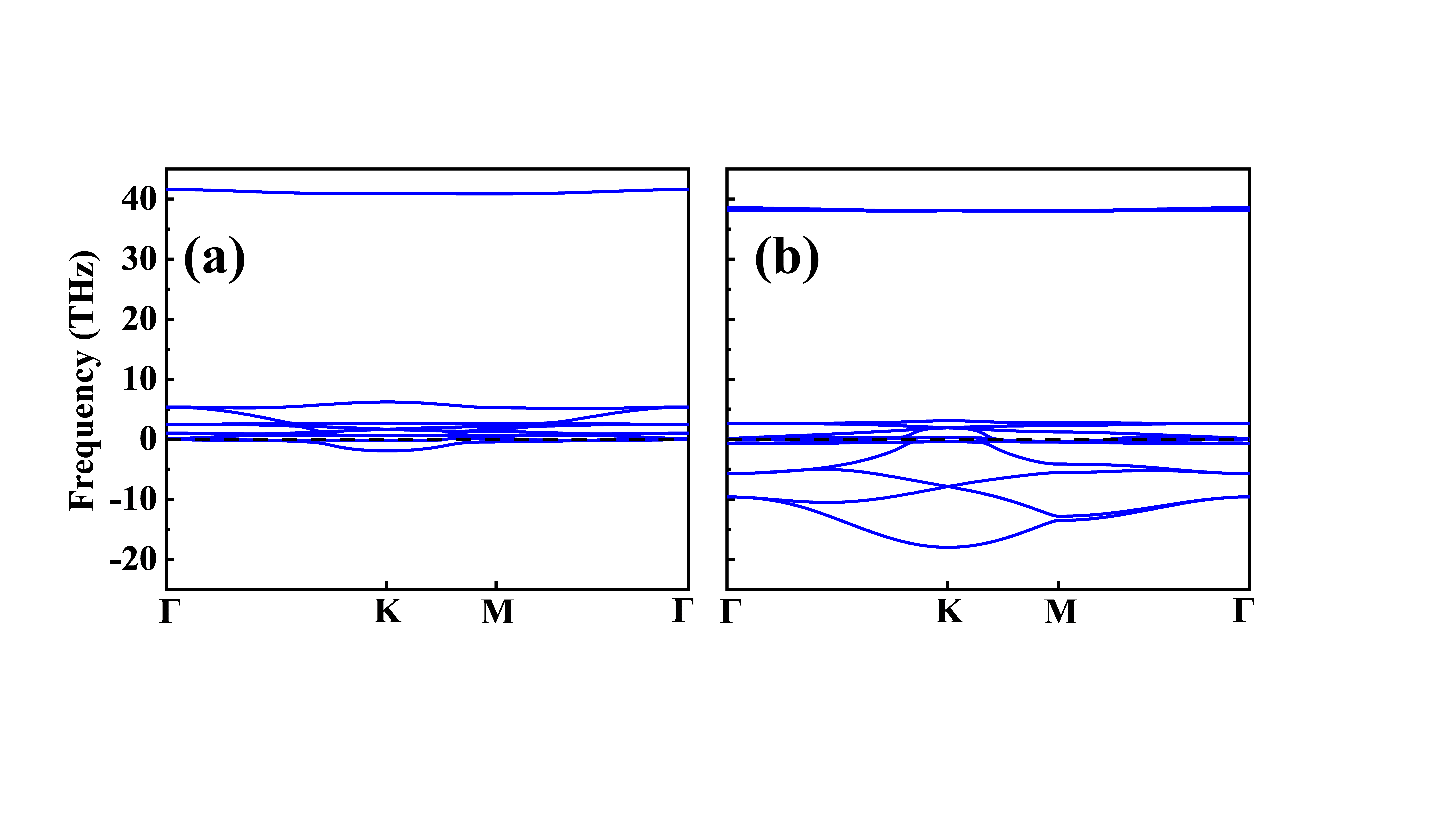


**Fig. S1.** The calculated phonon spectra of the Tl_2_H (a) and Tl_2_H_2_ (b) monolayers.


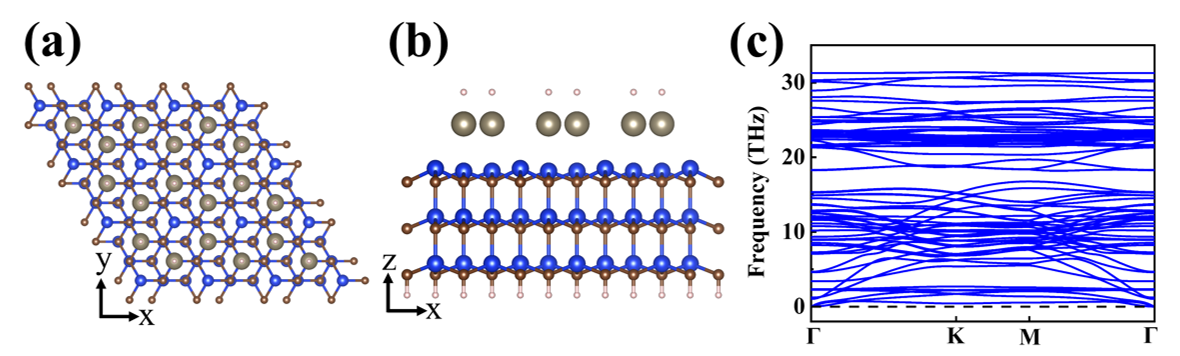


**Fig. S2.** The top (a) and side (b) views for the Tl_2_H_2_ monolayer on the SiC substrate. (c) The calculated phonon spectrum of the Tl_2_H_2_ monolayer on the SiC substrate.


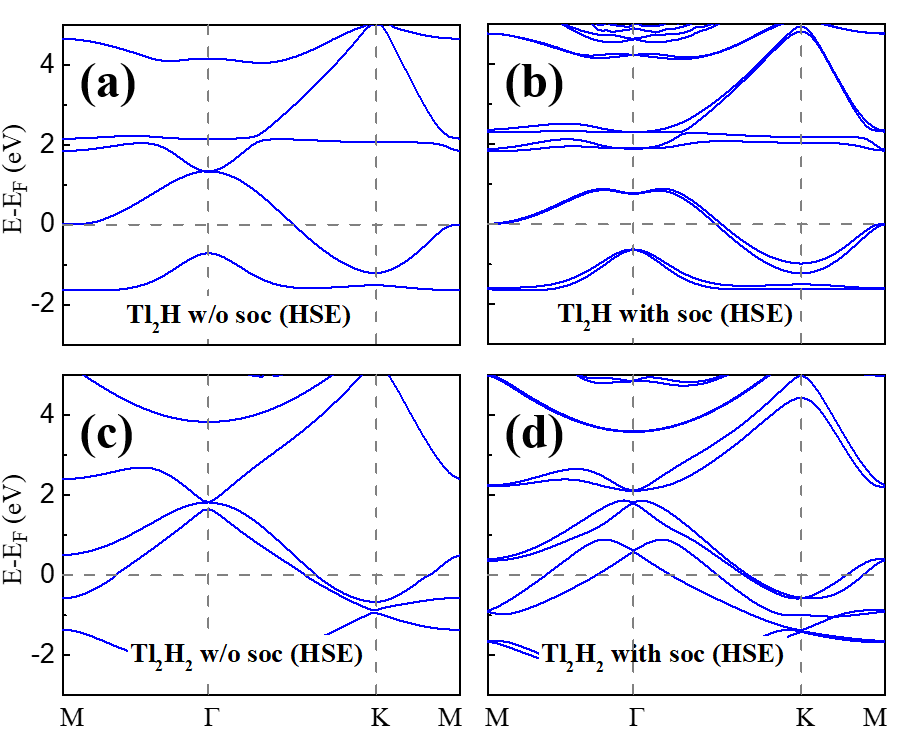


**Fig. S3.** Band structures obtained with HSE06 functional. (a) and (b) are for the Tl_2_H monolayer without and with SOC, respectively. (c) and (d) are for the Tl_2_H_2_ monolayer without and with SOC, respectively.

**Fig. S4.** The binding energies as a function of the strain for the monolayer Tl_2_H and Tl_2_H_2_. The red and green colors give the Tl_2_H and Tl_2_H_2_ results, respectively.

**
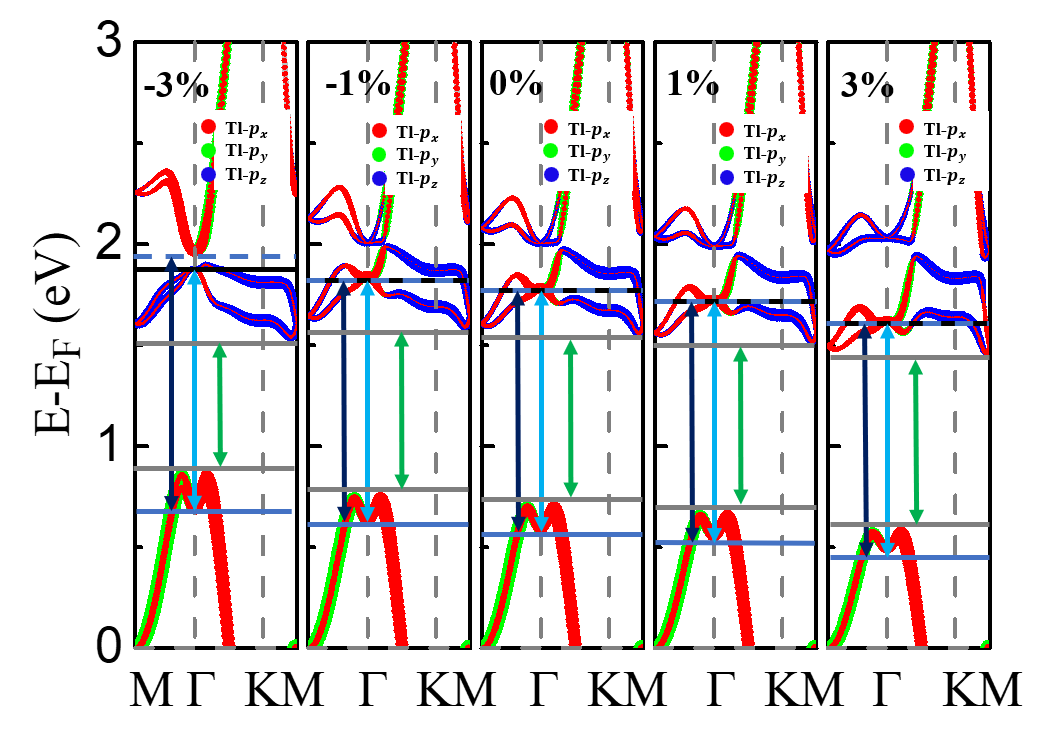
**

**Fig. S5.** (Color online) The orbital-projected band structures of the monolayer Tl_2_H under different strengths of the biaxial strain. The SOC is considered. The dot size is proportional to the contribution of the corresponding orbitals. The blue arrow represents the direct band gap (△E_d_) at the Γ point. The black arrow represents the split of *p_x/y_* orbitals for SOC. The green arrow represents the global band gap (△E_g_).


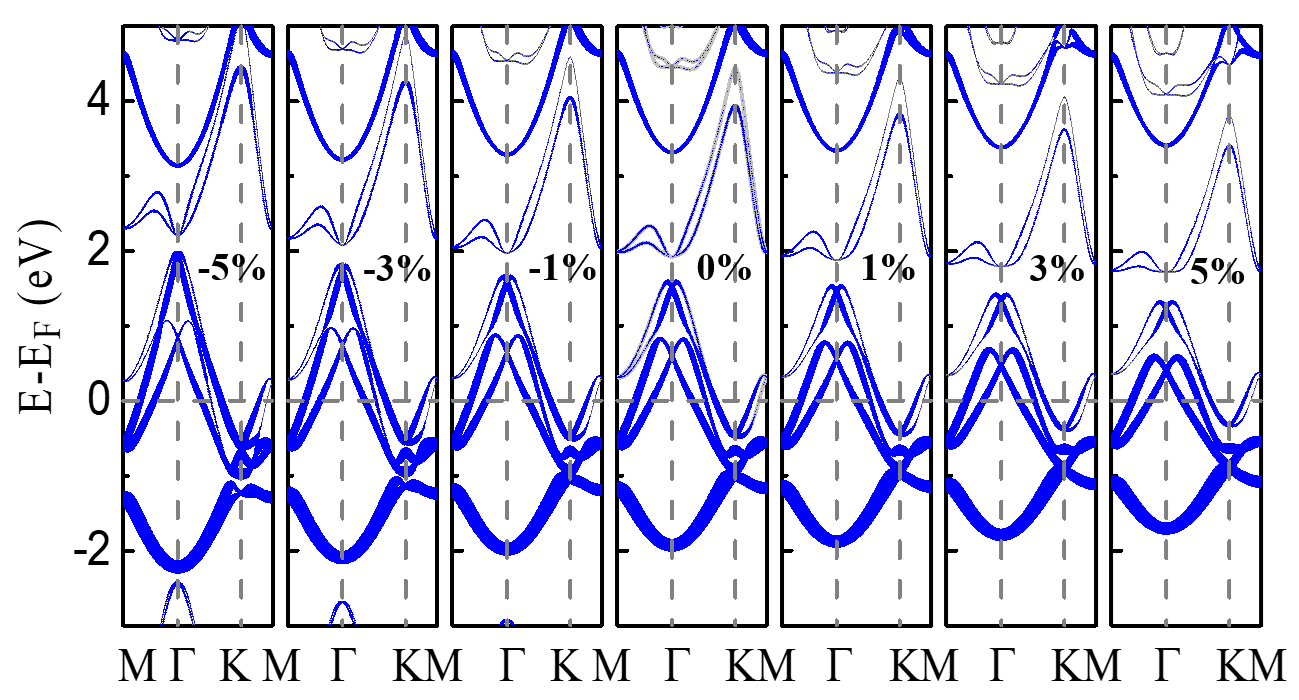


**Fig. S6.** (Color online) The band structures of the monolayer Tl_2_H_2_ under different strengths of the biaxial strain. The SOC is considered. The blue dots represent the distribution of *p_z_* orbital in the system under the corresponding conditions.
